# Genomic approach to determine sources of neonatal *Staphylococcus aureus* infection from carriage in the Gambia

**DOI:** 10.1186/s12879-024-09837-5

**Published:** 2024-09-09

**Authors:** Abdoulie Bojang, Matthew Chung, Bully Camara, Isatou Jagne, Romain Guérillot, Ebrahim Ndure, Benjamin P Howden, Anna Roca, Elodie Ghedin

**Affiliations:** 1 Systems Genomics Section, Laboratory of Parasitic Diseases, National Institute of Allergy and Infectious Diseases, NIH, Bethesda, MD USA; 2 MRC Unit The Gambia at the London School of Hygiene and Tropical Medicine, Fajara, The Gambia; 3 Department of Microbiology and Immunology, Doherty Institute, University of Melbourne, Melbourne, Australia; 4 Microbiological Diagnostic Unit Public Health Laboratory, Department of Microbiology and Immunology, Doherty Institute, University of Melbourne, Melbourne, Australia; 5 Centre for Pathogen Genomics, University of Melbourne, Melbourne, Australia

**Keywords:** Staphylococcus, Sequence types, Antimicrobial resistance, Virulence, The Gambia

## Abstract

*Staphylococcus aureus* is a major cause of neonatal infections in various anatomical sites, resulting in high morbidity and mortality in The Gambia. These clinical infections are often preceded by nasal carriage of *S. aureus*, a known risk factor. To determine whether potential sources of newborn *S. aureus* infections were from carriage, and to characterize *S. aureus* present in different anatomical sites (blood, ear, eye, umbilical cord, skin, pus, oropharynx, breast milk and vagina), we performed whole-genome sequencing of 172 isolates from clinical sites as well as from healthy and unhealthy carriage. A random selection of mothers (*n* = 90) and newborns (*n* = 42) participating in a clinical trial and testing positive for *S. aureus* were considered for this study. Sequence data were analyzed to determine *S. aureus* multilocus sequence types and selected antimicrobial and virulence gene profiles. Our findings revealed that in The Gambia, ST15 is the dominant sequence type associated with both carriage and clinical infection. In addition, *S. aureus* isolates causing clinical infection among neonates were genetically similar to those colonizing their oropharynx, and the different anatomical sites were not found to be uniquely colonized by *S. aureus* of a single genomic profile. Furthermore, while *S. aureus* associated with clinical infection had similar antimicrobial resistance gene profiles to carriage isolates, only hemolysin and adhesive factor virulence genes were significantly higher among clinical isolates. In conclusion, this study confirmed *S. aureus* oropharyngeal colonization among neonates as a potential source of clinical infection in The Gambia. Hence, interventions aiming to reduce neonatal clinical infections in The Gambia should consider decreasing oropharyngeal *S. aureus* carriage.

**Trial registration **The trial was registered at ClinicalTrials.gov NCT03199547.

**Supplementary Information:**

The online version contains supplementary material available at 10.1186/s12879-024-09837-5.

## Background

Globally, neonatal mortality remains a major public health concern. While mortality rates among children under five and 5–14 years declined by 59% and 53%, respectively, neonatal mortality, defined as death during the first 28 days of life, has remained unchanged [1, 2]. There exists a great disparity between low- and middle-income countries (LMICs), typically in Southern Asia and Sub-Saharan Africa (SSA), compared to high-income countries, with the former recording mortality rates at least ten times higher than those in high-income countries [3]. Hence, there is an urgent need to reduce these unacceptably high numbers of neonatal deaths.

The United Nations Sustainable Development Goal (SDG) number 3 calls for an end to preventable deaths of neonates, with all countries aiming to reduce neonatal mortality to at least 12 deaths per 1,000 live births by 2030 [4]. In The Gambia, neonatal mortality was reported to be 26 per 1000 live births [1]; severe infections, mainly sepsis, remained one of the leading causes of death [5, 6]. The most common bacterial cause of these severe neonatal infections, especially during the first week of life, is *Staphylococcus aureus* [7]. Bacterial infection could potentially be acquired from the birth canal during delivery or in the first days of life through close physical contact with the mother, as bacterial colonization in mothers is especially common in resource-limited settings, including SSA [8]. Infection may also be acquired from environmental sources, especially under poor hygiene conditions [9].

Bacterial infection is often preceded by carriage, and nasal carriage of *S. aureus* is a major risk factor for invasive *S. aureus* disease. Neonatal nasopharyngeal carriage of *S. aureus* begins a few days after birth and peaks at approximately 3 weeks of age [10]. Among Gambian newborns, *S. aureus* nasopharyngeal carriage prevalence was reported to range between 50 and 63% [10, 11]. Neonatal nasopharyngeal carriage of *S. aureus* was found to be significantly associated with maternal carriage in the breast milk, vaginal tract, and nasopharynx [12]. Oropharyngeal carriage prevalence was reported to reach 65% among infants [13].

The population structure of *S. aureus* comprises clonal complexes (CCs) that differ in their genetic makeup. These CCs can be identified by multilocus sequence typing (MLST), which detects polymorphisms in several housekeeping genes [14]. Different sequence types carry unique accessory gene regulator *agr* alleles in the *agr* operon [15], which might determine the successful colonization of particular anatomical sites.

Data on the genomic identity of carriage and clinical isolates of *S. aureus* from the same individual have been conflicting, often limited by study designs challenged by sample collection and clonal diversity. There are reports that progression from carriage to clinical infection is often associated with the acquisition of new virulence determinants or mutations in the *agr operon* [16, 17]. However, other studies found no association between invasiveness and core or accessory gene content or variation in *S. aureus* [18].

In this study, we aimed to determine the genetic relatedness between clinical and carriage isolates of *S. aureus* from neonates in a clinical trial (PregnAnZI − 2 trial) to establish oropharyngeal carriage as a potential source of newborn clinical infection. We also analyzed the genetic profile of *S. aureus* isolates from different anatomical sites among mothers and newborns to determine whether a specific biological niche is uniquely colonized by *S. aureus* of a particular genomic profile.

## Materials and methods

### PregnAnZI-2 trial

This study leverages samples from a phase III, double-blinded, placebo-controlled randomized multicenter clinical trial, the details of which have already been published [19]. In brief, the trial involved approximately 12,000 pregnant women aged at least 16 years who gave consent and were present at the study health facilities during labor in The Gambia and Burkina Faso. The women were randomized to receive either a single oral dose of 2 g of oral azithromycin or placebo. Following the intervention, both mother and newborn pairs were followed-up at 28 days postdelivery to assess health and mortality. Passive visits were conducted to collect adverse events, including hospitalizations. Clinical samples, including blood cultures, umbilical cord swabs, skin swabs, eye swabs, and ear swabs, were also collected for assessment of neonatal and puerperal infections, including sepsis. A clinical infection is defined by observable signs and symptoms in a patient. For the samples analyzed, the term “clinical” refers to *S. aureus* isolates from newborns with clinical infection, “unhealthy” refers to carriage isolates of *S. aureus* obtained from the oropharynx of newborn with clinical infection, and “healthy” refers to carriage isolates of *S. aureus* from healthy mothers with no clinical infection.

In the carriage substudy, a cohort of 250 mother and newborn pairs per country were included to assess colonization. Mothers had breast milk and rectovaginal samples collected, while the newborns had rectal swabs. Both the mother and newborn had nasopharyngeal and oropharyngeal swabs collected.

### Study design

In this post hoc study, only study participants from The Gambia were included. Women were recruited in a peri-urban region from two government health facilities, Bundung Maternal and Child Health Hospital (BMCHH) and Serekunda Health Centre. These two hospitals are largely representative as women travel from all over the country to deliver in these hospitals. Babies with clinical infection who had *S. aureus* identified as the cause of infection and a subset of these babies who also carry *S. aureus* in the oropharynx were considered. We also randomly selected a number of healthy mothers who were found to carry *S. aureus* in their oropharynx, breast milk, and vaginal tract.

### Participant and sample selection

Participants (*n* = 42) from the Gambian arm of PregnAnZI-2 exhibiting clinical symptoms who underwent sample collection including blood cultures, eye swabs, skin swabs, umbilical cord swabs, and abscess swabs, and that were identified as positive for *S. aureus*, were chosen for inclusion in this study. Furthermore, participants (*n* = 5) with clinical symptoms who also carried *S. aureus* in the oropharynx were included. None of the participants with ear infections in the trial also carried *S. aureus* in the oropharynx. Except for oropharyngeal carriage, only one sample per study subject (both mothers and babies) were collected. For each oropharyngeal sample, up to 8 suspected colonies of *S. aureus* were screened to account for the genetic diversity that may exist in the oropharynx. In addition, a random selection of oropharyngeal, breast milk and rectovaginal samples positive for *S. aureus* from healthy study participants (*n* = 90) was included. Of note, none of the 90 mothers are biologically related to the babies with clinical *S. aureus* infections. All samples (*n* = 172) were collected between November 2017 and April 2021. The flow chart (Supplemental Fig. 1) shows the selection process for the study.

### Culture, DNA extraction, and sequencing

To determine the genetic relatedness between clinical and carriage *S. aureus* isolates, we performed whole-genome sequencing. Frozen vials containing isolates of *S. aureus* in 80% glycerol from the PregnAnZI-2 trial were scraped with a sterile loop and streaked on blood agar plates (BAP) to obtain discrete colonies. A single isolated colony was confirmed as *S. aureus* using the Staphaurex™ Latex Agglutination Test kit (Thermo Scientific™, UK cat no. R30950102). Oropharyngeal swabs in skim milk, tryptone, glucose, and glycerin (STGG) were initially thawed, vortexed briefly before 10 µl was dispensed onto mannitol salt agar plates and incubated at 35 °C for 48 h. Up to 8 colonies of suspected *S. aureus* were subcultured on blood agar plates (BAP) before being confirmed with the Staphaurex™ Latex Agglutination Test kit. A total of 172 *S. aureus* isolates from 132 study participants were included. Genomic DNA was extracted from confirmed *S. aureus* isolates using the QIAGEN QIAamp DNA Mini Kit, UK following the manufacturer’s protocol. DNA sequencing libraries were prepared using the Nextera XT kit (Illumina, San Diego, CA, United States) and sequenced on the Illumina MiSeq platform using 2 × 150-bp chemistry.

### Data analysis

Illumina paired-end reads were initially trimmed for adapter sequences using Trimmomatic (v.0.36) [20]. The paired-end reads were assembled using the SKESA portion of the RAPT pipeline (v0.5.1) [21] using “*Staphylococcus aureus*” as the designated organism. Protein-coding genes were called for each assembly using the PGAP portion of the RAPT pipeline. To assess the distribution of *S. aureus* sequence types among clinical and carriage isolates in our study, we used multilocus sequence typing (MLST). For each sample, the contigs were used to query the MLST database [22] to determine the alleles for each of the 7 housekeeping genes, a combination of which gives a sequence type (ST). The MEGARes v3.0 database was used for antimicrobial resistance and virulence gene quantification using the AMR^++^ pipeline [23]. The chi-square test or Fisher’s exact test was used to determine the relationship between categorical variables with a significance threshold of *p* < 0.05.

### Phylogenomics

The 156 *S. aureus* genomes successfully sequenced in this study along with 1157 publicly available ST15 *S. aureus* genomes downloaded from the Staphopia database [24] were aligned onto the fully assembled reference genome of *S. aureus* 315 (*GCF_003354925.1*) using snippy (v4.6.0) [25]. As in Guérillot et al. [26], high-confidence variants were filtered by removing aligned reads having mapping quality below 60 and requiring a minimum depth of 20 reads with at least 90% of reads supporting the variant. To remove low-quality genome sequences, we only kept genomes for which 80% or more bases were aligned to the reference genome. The alignment of full genomes was filtered for SNP sites (v2.3.2) [27] to remove monomorphic sites and then trimmed with Trimal (v1.4.rev15) [28] to retain only alignment positions with less than 2% gaps or ambiguous positions. Maximum likelihood phylogeny was inferred from the resulting core genome variant alignment with FastTree (v2.1.8) [29] using the generalized time-reversible (GTR) model. Contextualization involved initial down-sampling keeping only publicly available ST15 strains closest to the study ST15. Tree plots were generated with ggtree version 3.8.2. R package.

## Results

To determine the genetic relatedness between clinical and carriage isolates of *S. aureus* from neonates and the genomic profile of *S. aureus* isolates collected from different anatomical sites among mothers and neonates, we performed whole-genome sequencing on 172 isolates. Of the total, 8 samples failed due to low DNA concentration, and another 8 were excluded following assembly as the contigs generated did not meet the threshold of 5 kb. Twenty-one (13%) isolates that had tested positive for *S. aureus* using the agglutination assay were found by whole genome sequencing to be species other than *S. aureus* (Suppl. Table 1).

### ***S. aureus*** sequence type 15 dominates in The Gambia

**Fig. 1:**
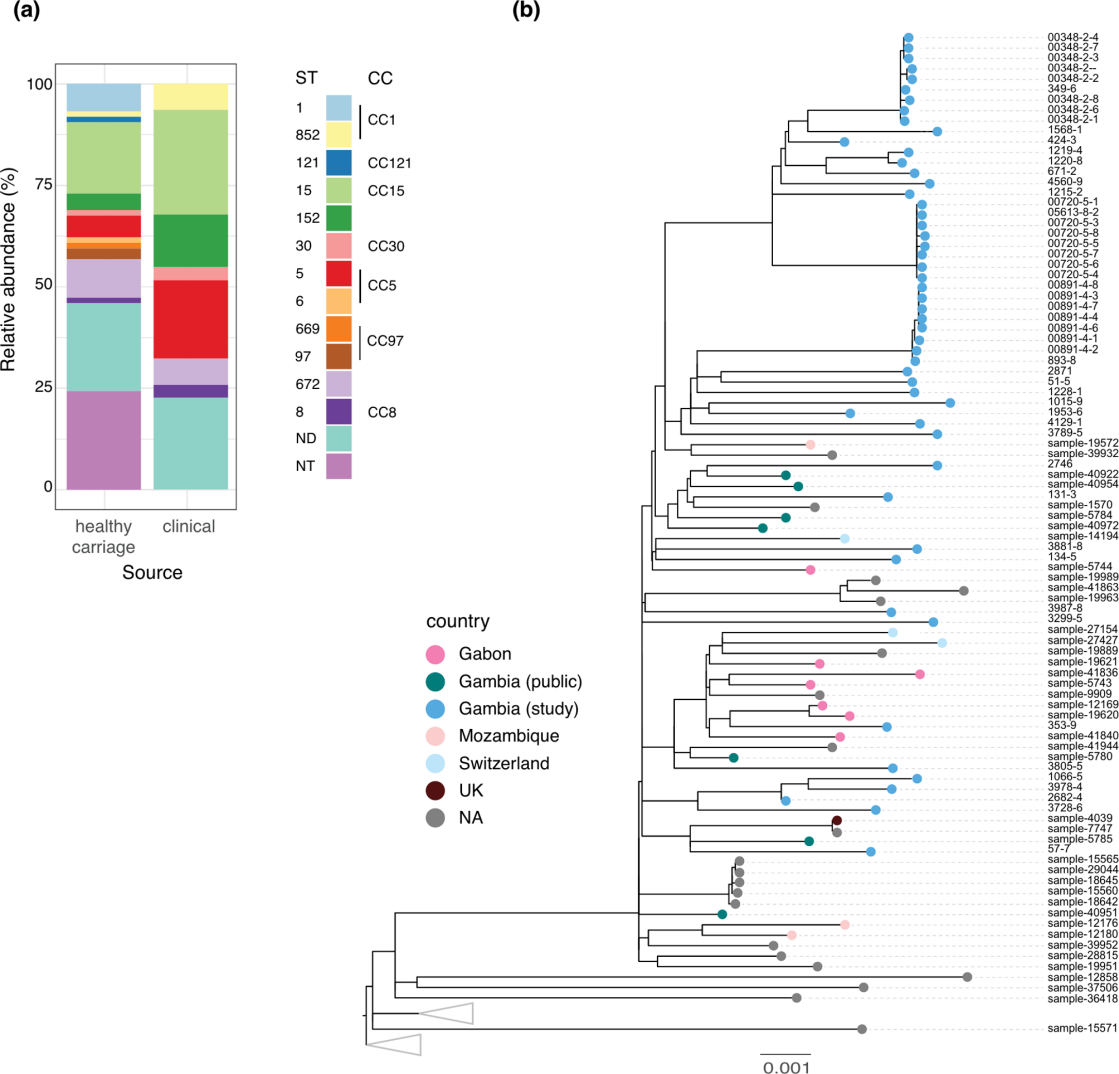
**(a)** Stacked bar chart showing the relative abundance as percentages of sequence types among healthy carriage (*n* = 74) and clinical samples (*n* = 31). ST = sequence type, CC = clonal complexes, ND = not determined, NT = not typable. **(b)** Gambian cluster from a maximum likelihood phylogenetic tree (Supp Fig. 2) showing the evolutionary relationship between Gambian ST15 and publicly available ST15s globally. Scale 0.001 (~ 60 substitutions). NA = isolates of *S. aureus* in the Staphopia database with country of origin not indicated. UK = United Kingdom. Grey triangles represent collapsed branches from the global tree

To assess the distribution of *S. aureus* STs among clinical and carriage isolates in our study, we used multilocus sequence typing. In this analysis, only one isolate per individual was included except in one instance where a participant was colonized by two different sequence types. The distribution of the *S. aureus* samples analyzed (*n* = 133) revealed thirteen different sequence types (STs) belonging to 8 clonal complexes (CC). The four most frequent STs were ST15 (28%), ST672 (10%), ST852 (7%), and ST5 (6%). There was higher diversity of sequence types among healthy carriage (*n* = 12) compared to clinical (*n* = 7) samples, with ST15 being dominant in both (clinical = 25.8% and carriage = 23.2%) (Fig. 1a).

To investigate the evolutionary relationship among the dominant ST15 with those of other ST15 reported globally (*n* = 1157), we performed a phylogenetic analysis using the maximum likelihood method. Our findings revealed unique clustering of Gambian ST15 from the global collection (Fig. 1b and Supp. Figure 2), except for one Gambian sample (826-1), indicating that Gambian ST15 samples share a common ancestor, likely reflecting recent or ongoing transmission.

### Diversity of ***S. aureus*** sequence types found across body sites

*S. aureus* is known to colonize or infect various parts of the human body. We assessed the association of *S. aureus* STs from different sample types to determine whether an ST could be overrepresented in a particular biological niche. Our findings revealed that the same *S. aureus* STs were found in the oropharynx, skin, abscess, and rectovaginal samples (Supp. Figure 3, Suppl. Table 2). Furthermore, we assessed the proportion of dominant STs in clinical and carriage samples to determine whether some STs were disproportionally associated with clinical infections. Fisher’s exact test showed a significant association between the proportion of ST5 in clinical samples and carriage (4.43 95%CI 1.51–15.85 *p* = 0.004). No significant association was observed for the other dominant STs, ST15 (1.60 95% CI 0.77–3.36 *p* = 0.232), ST672 (0.58 95%CI 0.17–1.83 *p* = 0.435) and ST852 (6.27 95% CI 0.74–293.12 *p* = 0.118). ST 1, 6, 97, 121 and 669 only occurred in the healthy carriage category, but the numbers were too small (≤ 4 isolates) to conclude that they were enriched in carriage compared to clinical samples. Furthermore, ST672 (0 95% CI 0–0.29 *p* < 0.001), ST852 (0 95% CI 0–0.48 *p* = 0.003) and ST5 (7.04 95% CI 1.95–38.56 *p* < 0.001) were found to be associated with azithromycin exposure. This was not the case for ST15 (0.51 95% CI 0.25–1.05 *p* = 0.07).

### Source of ***S. aureus*** infection

**Fig. 2:**
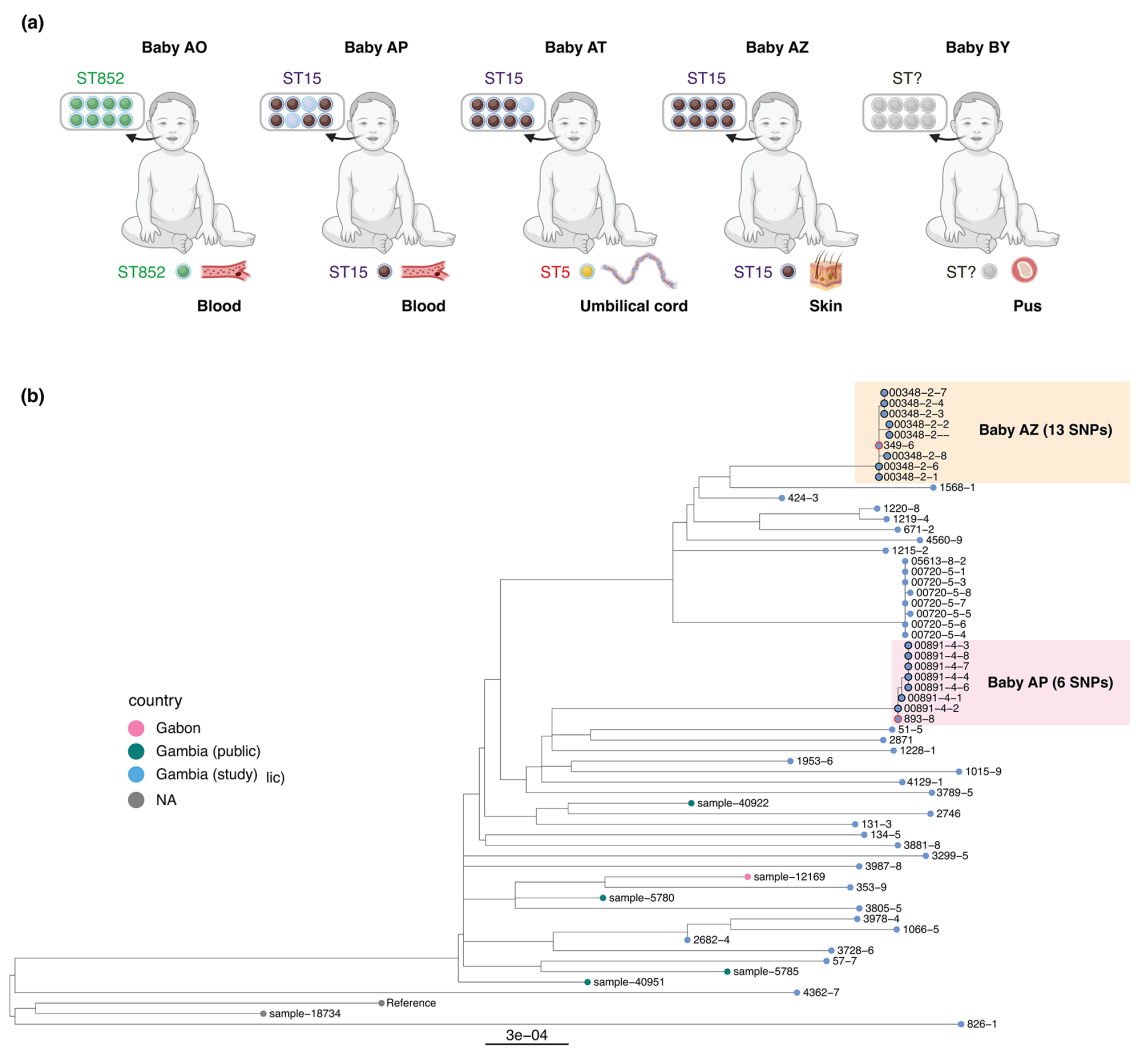
(**a**) Comparison of *S. aureus* STs from clinical (blood, pus, umbilical cord, and skin swabs) and carriage (oropharynx) isolates of the same patient (baby). (**b**) Phylogenetic tree showing ST15s from the same patient more closely related than those from other patients in the same study or a down-sample of publicly available ST15s globally. Red outline = clinical, black outline = carriage isolate from the same patient; colored boxes highlight samples from individual babies. Panel (**a**) was created with BioRender.com

Oropharyngeal carriage of *S. aureus* is a known risk factor for *S. aureus* clinical infection. We assessed the genetic relatedness of carriage and clinical isolates of *S. aureus* from neonates by multilocus sequence typing (MLST) to determine the potential source of *S. aureus* clinical infection. Of the 5 neonates for which we had clinical and unhealthy (i.e. associated with clinical infection) carriage samples, a range of different STs could be found. The comparison of clinical and carriage isolates from the same study patient revealed that, in four of the five cases, the *S. aureus* isolates associated with clinical infection in the blood, skin and abscess (pus) were of the same ST as those colonizing the oropharynx (Fig. 2a). The core genome of *S. aureus* from two patients with ST15 infections in the blood and skin was compared to their oropharyngeal carriage isolates. The results show that ST15 recovered from blood differed by only 6 SNPs from one of the oropharyngeal carriage isolates, while a difference of 13 SNPs was observed for the skin isolates (Fig. 2b), indicating that the oropharynx could have seeded both the invasive and skin infections. In contrast, the clinical isolate associated with infection of the umbilical cord was different from any of the STs colonizing the neonate’s oropharynx. The STs of 9 isolates from three of the babies could not be determined; for two of the babies (AP and AT), this was due to low coverage over a single allelic position *aroE*. The isolates, however, shared the same alleles with ST15 for the remaining allelic positions, suggesting that they may be of the same ST. In the case of the third baby (BY), all isolates have a *glpF* allele that is not in the MLST database, indicative of a novel ST.

### Antibiotic resistance and virulence genes

**Fig. 3:**
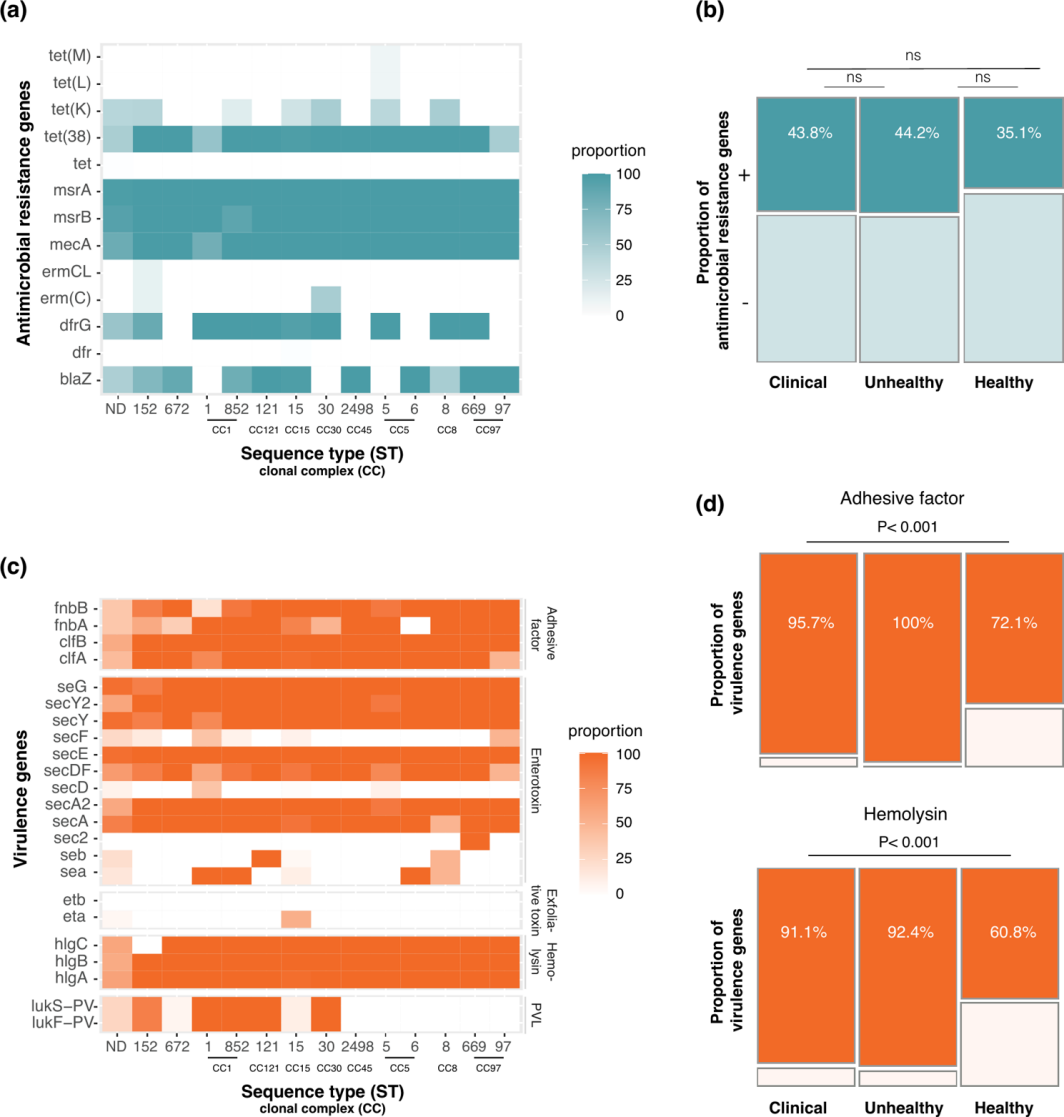
**(a)** Heatmap showing the proportion of antimicrobial resistance genes for the different sequence types. Genes associated with resistance to tetracycline (*tet(M)*, *tet(L)*, *tet(K)*, *tet(38)*, tet); β-lactams (*blaZ*, *mecA*), macrolides (*msrA*, *msrB*, *ermC*, *ermCL*), trimethoprim (*dfr*). **(b)** Mosaic plot showing the proportion of antimicrobial resistance genes among the three sample categories. **(c)** Heatmap showing the proportion of virulence genes among *S. aureus* STs. Genes associated with the adhesive factor (*fnbB*, *fnbA*, *clfB*, *clfA*), enterotoxin (*seG*, *secY2*, *secY*, *secF*, *secE*, *secDF*, *secD*, *secA2*, *secA*, *sec2*, *seb*, *sea*), exfoliative toxin (*etb*, *eta*), hemolysin (*hlgC*, *hlgB*, *hlgA*), Panton Valentine leukocidin PVL (*lukS-PV*, *lukF-PV*). **(d)** Mosaic plot showing the proportion of hemolysin and adhesive factor genes among the three sample categories

For more effective treatment of *S. aureus* infections, it is crucial to determine the antibiotic resistance profile of isolates. We screened all isolates for the presence of common antimicrobial resistance genes associated with resistance to penicillin (*blaZ*), cotrimoxazole (*dfrG*,* dfr*), methicillin (*mecA*), tetracycline (*tet*,* tet(38)*,* tet(K)*,* tet(L)*,* tet(M)*) and macrolides (*msrA*,* msrB*,* erm(C)*,* ermCL*). Our findings revealed that the presence of genes associated with antimicrobial resistance was significantly higher in *S. aureus* than in other species of staphylococci (*p* = 0.035, 41.4% vs. 26.5%). All 13 different *S. aureus* sequence types were found to carry at least one gene associated with penicillin, cotrimoxazole, tetracycline, or macrolide resistance, including a high prevalence of methicillin resistance as indicated by the presence of the mecA gene across the ST15 isolates (Fig. 3a). The proportion of *S. aureus* carrying any antimicrobial resistance gene was not significantly different between the three sample categories (43.8% vs. 44.2% vs. 35.1% for clinical, unhealthy, and healthy, respectively) (Fig. 3b). Furthermore, antibiotic exposure to azithromycin was not associated with the presence of any antimicrobial resistance gene (*p* = 0.899).

Differences in the presence or absence of selected *S. aureus* genes during a commensal lifestyle and as a pathogen may vary. To gain insight into the commensal-to-pathogen transition and how a bacterial pathogen adapts to different environments within the host, we screened for the presence of virulence genes of interest in all samples. Among all staphylococcus species identified in our study, *S. aureus* exhibited the highest prevalence of virulence-associated genes (*p* < 0.001), including hemolysin (*hlgA*, *hlgB*, *hlgC*) and adhesive genes (*fnbA*, *fnbB*, *clfA*, *clfB*). All 13 different *S. aureus* STs were found to carry at least one enterotoxin (*sea*,* seb*,* sec2*,* secA*,* secA2*,* secD*,* secDF*,* secE*,* secF*,* secY*,* secY2*,* seG*), adhesive factor and hemolysin virulence genes, whereas only a few STs carried the PVL (*lukS-PV*,* lukF-PV*) and exfoliative toxin genes (*eta*,* etb*) (Fig. 3c). Additionally, the proportion of virulence genes among the samples across 13 different *S. aureus* STs was similar for each of the gene classifications investigated (Fig. 3c). The prevalence of hemolysin (95.7% vs. 72.1%) and adhesive factor (91.1% vs. 60.1%) virulence genes was significantly higher among clinical isolates than among healthy carriage samples (*p* < 0.001; Fig. 3d). Furthermore, antibiotic exposure was significantly associated with both adhesive gene (*p* = 0.002) and hemolysin gene (*p* < 0.001) presence but not any other virulence gene categories (enterotoxin gene *p* = 0.532, exfoliative toxin *p* = 0.099, and PVL gene *p* = 0.102).

## Discussion

To our knowledge, this study is the first in The Gambia to investigate using a genomic approach both the potential source of neonatal *S. aureus* infection as well as the association of the genomic profile of *S. aureus* isolates to a particular anatomical site. This study confirms *S. aureus* ST15 in The Gambia as the dominant sequence type associated with both carriage and clinical infection with a high prevalence of antimicrobial resistance and virulence genes. It also shows oropharyngeal carriage as a potential source of clinical *S. aureus* infection among neonates. Interestingly, while *S. aureus* genomes associated with clinical infection had broadly similar antimicrobial resistance gene profiles to carriage isolates, they had significantly increased virulence gene profiles.

### ST15 dominance in The Gambia

Almost a third of all *S. aureus* isolates from mothers and newborns in this study belong to the ST15 clade. This is in line with previous reports of ST5 and ST15 dominance in both carriage [30] and clinical infection [31] in The Gambia. Interestingly, the dynamics appeared to have changed slightly in this study, with ST15 leading, followed by ST672 and a relatively smaller contribution from ST5. There are also reports of ST15 dominance in other parts of Africa, including Ghana, Nigeria [32, 33] and other countries [34, 35]. Surprisingly, almost all the ST15 isolates carried the mecA gene, indicating a high prevalence of methicillin resistance in the population. Methicillin resistance among *S. aureus* isolates was reported to be low from previous studies in The Gambia [36, 37].

### Association of S. aureus STs with sample type or category

This study did not find any unique association of *S. aureus* sequence type with a particular biological niche. Contrary to our findings, a study [38] using a genomic profiling method involving repetitive sequence PCR (repPCR) reported unique colonization of the anterior nares, axillae, and inguinal folds by specific *S. aureus* strain types. The difference in findings could be due to the higher discriminatory index of repPCR (D = 0.88) compared to MLST (D = 0.84) [39].

### Association between clinical and carriage isolates from the same patient

We have shown that clinical isolates of *S. aureus* from neonates with blood or skin infections were genetically similar to isolates colonizing their oropharynx. The clinical isolates were of the same sequence type with at least one of the carriage isolates, and whole genome comparison revealed only 6–13 SNP differences between isolates. Since carriage is a known risk factor for clinical infection, it is reasonable to infer that the colonizing isolates resulted in clinical infections among these neonates. Furthermore, the clinical isolates had similar antimicrobial and virulence gene profiles to the carriage isolates. This aligns with a recent study in China where more than 86% of *S. aureus* colonizing the anterior nares of children and their corresponding non-nasal clinical isolates were indistinguishable in *mecA*, PVL, and ST expression [40].

### Pattern of antimicrobial and virulence genes

Although *S. aureus* isolates from healthy individuals had a lower proportion of any antimicrobial resistance genes, it was not significantly different compared to clinical or unhealthy carriage isolates. Patients with clinical infection in the hospital or community are more likely to be exposed to antibiotics for treatment, resulting in a high prevalence of antimicrobial resistance genes. However, antibiotics could be accessed even without prescription in Gambia, resulting in high antibiotic misuse in the community. The uncontrolled use of antibiotics in communities probably explains the relatively high antibiotic resistance gene prevalence among healthy individuals. Interestingly, contrary to the report that no core or accessory gene content or variation of *S. aureus* is associated with invasiveness [18], this study found a significantly higher proportion of virulence genes among patients with a clinical condition compared to healthy volunteers, hence supporting the alternative hypothesis that the transition from a commensal to a pathogen lifestyle requires changes in the virulence gene profile.

### Limitations of the study

A few limitations apply to this study. First, a small proportion of the isolates were not *S. aureus* and were not included in some of the analyses. This is unlikely to affect the outcome of the investigation as the numbers were small (21 out of 172 isolates tested). Second, due to low coverage at allelic position *aroE*, the sequence type for 2 isolates could not be determined even though this was a rare occurrence. Third, only 5 patients met the criteria allowing their samples to be included in the comparison of clinical vs. carriage isolates from the same patient. While we observed similar STs in the clinical and carriage isolates in the same patients, most of these STs were ST15, which was the predominant type circulating. Consequently, although our findings indicate that carriage isolates are the likely source of clinical infection, the size limitation of our study prevents us from ruling out other potential sources, such as other close contacts to the patients or the environment, as no sample was collected from these other sources.

## Conclusion

This study confirmed the dominance of ST15 in The Gambia and the risk of neonatal *S. aureus* infection by colonizing strains of the oropharynx. Furthermore, *S. aureus* infection was associated with a higher proportion of virulence genes but not antimicrobial resistance genes. Prospective studies collecting samples from newborns, close contacts and the environment are needed to determine with higher accuracy the sources of *S. aureus* infections among newborns.

## Electronic supplementary material

Below is the link to the electronic supplementary material.

Supplementary Material 1

Supplementary Material 2

Supplementary Material 3

Supplementary Material 4

Supplementary Material 5

Supplementary Material 6

## Data Availability

Sequence data that support the findings of this study have been deposited in GenBank under BioProject PRJNA1010232.
