# Supplementary figures and images for: Genomic approach to determine sources of neonatal Staphylococcus aureus infection from carriage in the Gambia
Source: BMC Infect Dis. 2024 Sep 9;24:941. doi: 10.1186/s12879-024-09837-5 (PMC11384681; doi:10.1186/s12879-024-09837-5)

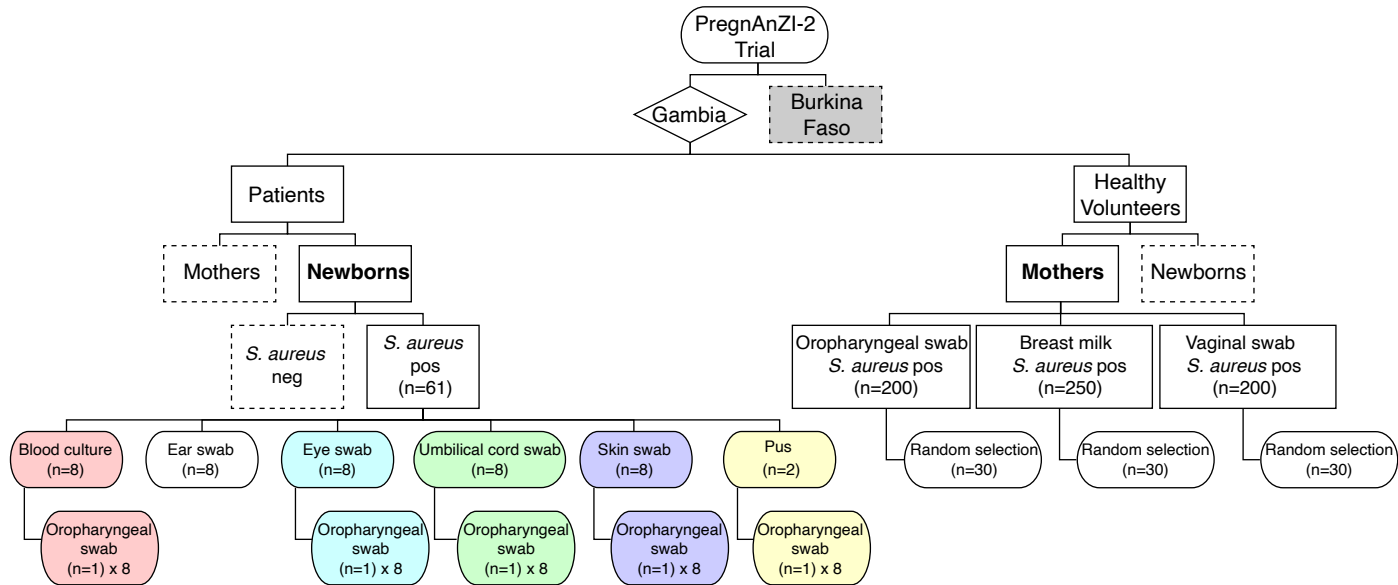

Supplement: Supplementary file 1 — Supplementary Material 1 [file 12879_2024_9837_MOESM1_ESM.pdf]

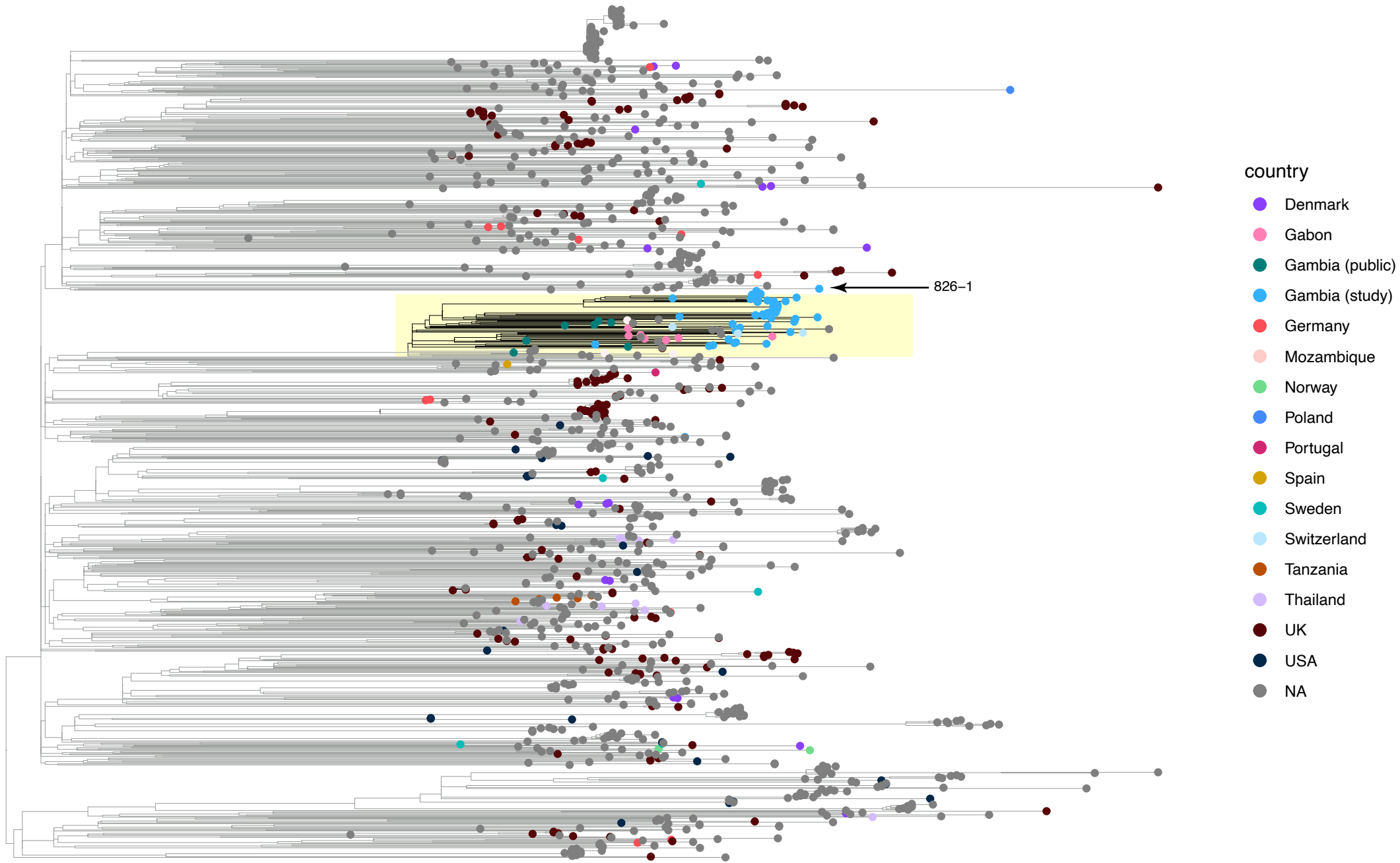

5e-04

Supplement: Supplementary file 2 — Supplementary Material 2 [file 12879_2024_9837_MOESM2_ESM.pdf]

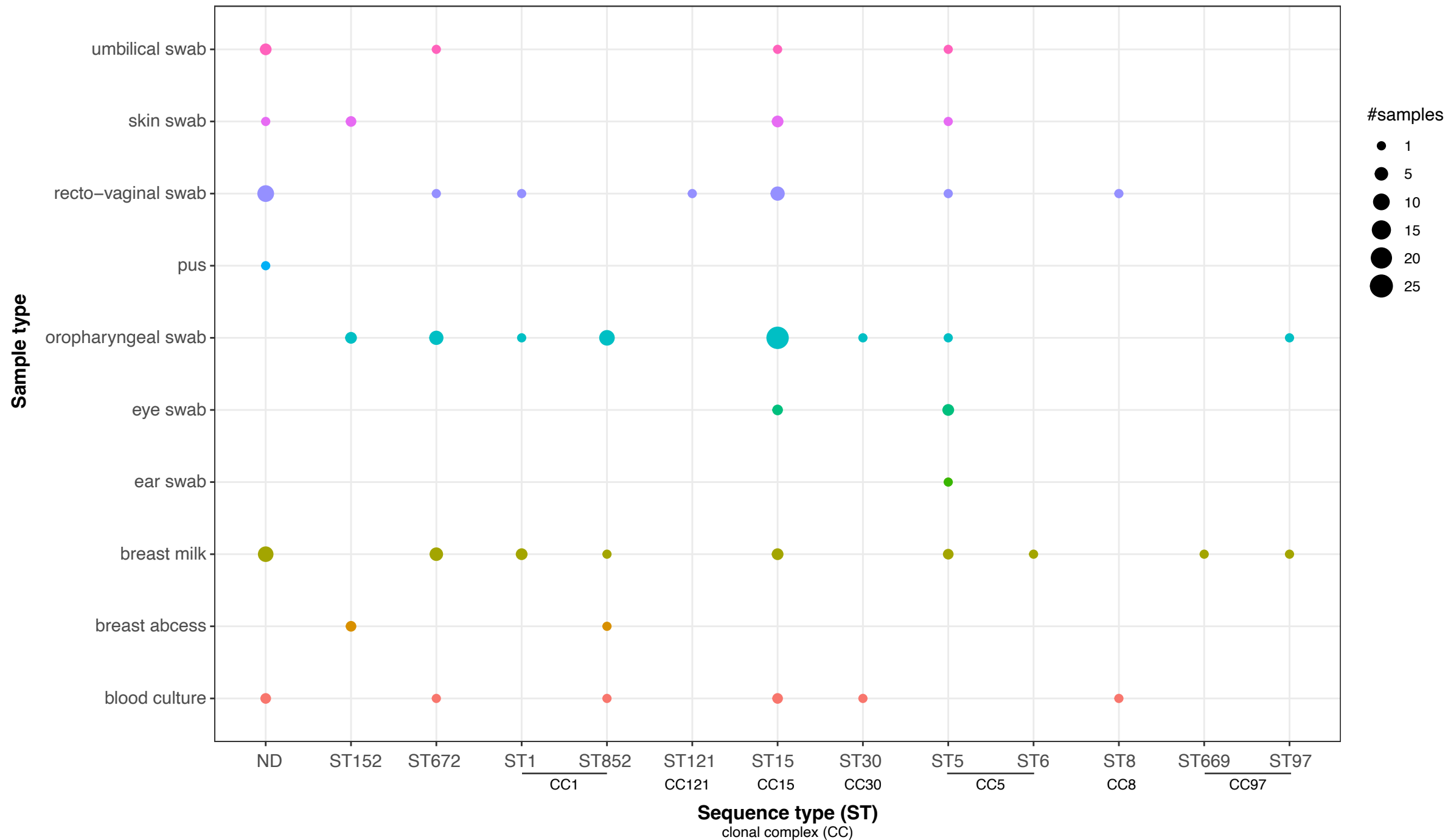

Supplement: Supplementary file 3 — Supplementary Material 3 [file 12879_2024_9837_MOESM3_ESM.pdf]
