# Supplementary material for: Genomic approach to determine sources of neonatal Staphylococcus aureus infection from carriage in the Gambia
Source: BMC Infect Dis. 2024 Sep 9;24:941. doi: 10.1186/s12879-024-09837-5 (PMC11384681; doi:10.1186/s12879-024-09837-5)
Supplement: Supplementary file 4 — Supplementary Material 4 [file 12879_2024_9837_MOESM4_ESM.pdf]

Suppl. Table 1. List of staphyloccoci species among study participants

Sample.no.	Patient	Category	Sample type	Sample.category	Species	Sequence type
1007	BC	Azithromycin	oropharyngeal swab	healthy carriage	*Staphylococcus aureus*	97
2266	BV	Azithromycin	oropharyngeal swab	healthy carriage	*Staphylococcus arlettae*	NA
2373	BX	Placebo	oropharyngeal swab	healthy carriage	*Staphylococcus arlettae*	NA
2746	CE	Azithromycin	breast milk	healthy carriage	*Staphylococcus aureus*	15
2767	BT	Placebo	breast milk	healthy carriage	*Staphylococcus aureus*	1
2871	CI	Placebo	breast milk	healthy carriage	*Staphylococcus aureus*	ND
2949	CM	Azithromycin	recto-vaginal swab	healthy carriage	*Staphylococcus aureus*	ND
3636	CZ	Azithromycin	oropharyngeal swab	healthy carriage	*Staphylococcus ureilyticus*	NA
3768	BZ	Azithromycin	breast milk	healthy carriage	*Staphylococcus kloosii*	NA
00348-2--	n/a	n/a	n/a	n/a	*Staphylococcus aureus*	15
00348-2-1	AP	Placebo	oropharyngeal swab	unhealthy carriage	*Staphylococcus aureus*	15
00348-2-2	AP	Placebo	oropharyngeal swab	unhealthy carriage	*Staphylococcus aureus*	ND
00348-2-3	AP	Placebo	oropharyngeal swab	unhealthy carriage	*Staphylococcus aureus*	15
00348-2-4	AP	Placebo	oropharyngeal swab	unhealthy carriage	*Staphylococcus aureus*	15
00348-2-6	AP	Placebo	oropharyngeal swab	unhealthy carriage	*Staphylococcus aureus*	ND
00348-2-7	AP	Placebo	oropharyngeal swab	unhealthy carriage	*Staphylococcus aureus*	15
00348-2-8	AP	Placebo	oropharyngeal swab	unhealthy carriage	*Staphylococcus aureus*	15
00476-9-1	AO	Placebo	oropharyngeal swab	unhealthy carriage	*Staphylococcus aureus*	852
00476-9-2	AO	Placebo	oropharyngeal swab	unhealthy carriage	*Staphylococcus aureus*	852
00476-9-3	AO	Placebo	oropharyngeal swab	unhealthy carriage	*Staphylococcus aureus*	852
00476-9-4	AO	Placebo	oropharyngeal swab	unhealthy carriage	*Staphylococcus aureus*	852
00476-9-5	AO	Placebo	oropharyngeal swab	unhealthy carriage	*Staphylococcus aureus*	852
00476-9-6	AO	Placebo	oropharyngeal swab	unhealthy carriage	*Staphylococcus aureus*	852
00476-9-7	AO	Placebo	oropharyngeal swab	unhealthy carriage	*Staphylococcus aureus*	852
00476-9-8	AO	Placebo	oropharyngeal swab	unhealthy carriage	*Staphylococcus aureus*	852
00720-5-1	AT	Placebo	oropharyngeal swab	unhealthy carriage	*Staphylococcus aureus*	15
00720-5-3	AT	Placebo	oropharyngeal swab	unhealthy carriage	*Staphylococcus aureus*	15
00720-5-4	AT	Placebo	oropharyngeal swab	unhealthy carriage	*Staphylococcus aureus*	ND
00720-5-5	AT	Placebo	oropharyngeal swab	unhealthy carriage	*Staphylococcus aureus*	15
00720-5-6	AT	Placebo	oropharyngeal swab	unhealthy carriage	*Staphylococcus aureus*	15
00720-5-7	AT	Placebo	oropharyngeal swab	unhealthy carriage	*Staphylococcus aureus*	15
00720-5-8	AT	Placebo	oropharyngeal swab	unhealthy carriage	*Staphylococcus aureus*	15
00891-4-1	AZ	Placebo	oropharyngeal swab	unhealthy carriage	*Staphylococcus aureus*	15
00891-4-2	AZ	Placebo	oropharyngeal swab	unhealthy carriage	*Staphylococcus aureus*	15
00891-4-3	AZ	Placebo	oropharyngeal swab	unhealthy carriage	*Staphylococcus aureus*	15
00891-4-4	AZ	Placebo	oropharyngeal swab	unhealthy carriage	*Staphylococcus aureus*	15
00891-4-6	AZ	Placebo	oropharyngeal swab	unhealthy carriage	*Staphylococcus aureus*	15
00891-4-7	AZ	Placebo	oropharyngeal swab	unhealthy carriage	*Staphylococcus aureus*	15
00891-4-8	AZ	Placebo	oropharyngeal swab	unhealthy carriage	*Staphylococcus aureus*	15
02415-9-1	BY	Placebo	oropharyngeal swab	unhealthy carriage	*Staphylococcus aureus*	ND
02415-9-2	BY	Placebo	oropharyngeal swab	unhealthy carriage	*Staphylococcus aureus*	ND
02415-9-3	BY	Placebo	oropharyngeal swab	unhealthy carriage	*Staphylococcus aureus*	ND
02415-9-5	BY	Placebo	oropharyngeal swab	unhealthy carriage	*Staphylococcus aureus*	ND
02415-9-6	BY	Placebo	oropharyngeal swab	unhealthy carriage	*Staphylococcus aureus*	ND
02415-9-7	BY	Placebo	oropharyngeal swab	unhealthy carriage	*Staphylococcus aureus*	ND
02415-9-8	BY	Placebo	oropharyngeal swab	unhealthy carriage	*Staphylococcus aureus*	ND
05613-8-1	DW	Placebo	oropharyngeal swab	unhealthy carriage	*Staphylococcus aureus*	672
05613-8-2	DW	Placebo	oropharyngeal swab	unhealthy carriage	*Staphylococcus aureus*	15
05613-8-3	DW	Placebo	oropharyngeal swab	unhealthy carriage	*Staphylococcus aureus*	672
05613-8-4	DW	Placebo	oropharyngeal swab	unhealthy carriage	*Staphylococcus aureus*	672
05613-8-6	DW	Placebo	oropharyngeal swab	unhealthy carriage	*Staphylococcus aureus*	672
05613-8-7	DW	Placebo	oropharyngeal swab	unhealthy carriage	*Staphylococcus aureus*	672
05613-8-8	DW	Placebo	oropharyngeal swab	unhealthy carriage	*Staphylococcus aureus*	ND
1015-9	BD	Placebo	oropharyngeal swab	healthy carriage	*Staphylococcus aureus*	ND
1066-5	BB	Placebo	recto-vaginal swab	healthy carriage	*Staphylococcus aureus*	15
109-3	AD	Azithromycin	eye swab	clinical	*Staphylococcus aureus*	5
1215-2	BH	Azithromycin	oropharyngeal swab	healthy carriage	*Staphylococcus aureus*	15
1219-4	BI	Placebo	oropharyngeal swab	healthy carriage	*Staphylococcus aureus*	ND
1220-8	BI	Placebo	recto-vaginal swab	healthy carriage	*Staphylococcus aureus*	15
1228-1	BG	Azithromycin	oropharyngeal swab	healthy carriage	*Staphylococcus aureus*	15
1235-4	BF	Placebo	recto-vaginal swab	healthy carriage	*Staphylococcus aureus*	672
1248-5	BE	Azithromycin	oropharyngeal swab	healthy carriage	*Staphylococcus warneri*	NA
1257-6	BJ	Azithromycin	oropharyngeal swab	healthy carriage	*Staphylococcus aureus*	1
1258-4	BJ	Azithromycin	recto-vaginal swab	healthy carriage	*Staphylococcus aureus*	1
129-1	AF	Azithromycin	eye swab	clinical	*Staphylococcus aureus*	5
1290-7	BG	Azithromycin	oropharyngeal swab	healthy carriage	*Staphylococcus aureus*	5
1295-5	BF	Placebo	oropharyngeal swab	healthy carriage	*Staphylococcus aureus*	672
131-3	AH	Placebo	umblical swab	clinical	*Staphylococcus aureus*	15
1322-9	BM	Placebo	recto-vaginal swab	healthy carriage	*Staphylococcus aureus*	5
134-5	AI	Placebo	Blood culture	clinical	*Staphylococcus aureus*	15
1568-1	BQ	Azithromycin	recto-vaginal swab	healthy carriage	*Staphylococcus aureus*	15
1730-1	BO	Placebo	recto-vaginal swab	healthy carriage	*Mammaliicoccus sciuri*	NA
1784-3	BP	Azithromycin	breast milk	healthy carriage	*Staphylococcus aureus*	5
1841-1	n/a	n/a	n/a	n/a	*Staphylococcus saprophyticus*	NA
1890-2	BN	Placebo	breast milk	healthy carriage	*Staphylococcus aureus*	1
1907-5	BR	Placebo	breast milk	healthy carriage	*Staphylococcus aureus*	ND
1953-6	BW	Placebo	oropharyngeal swab	healthy carriage	*Staphylococcus aureus*	ND
2010-2	BU	Azithromycin	breast milk	healthy carriage	*Staphylococcus aureus*	852

2408-1	CA	Placebo	recto-vaginal swab	healthy carriage	*Staphylococcus aureus*	121
2418-4	CA	Placebo	breast milk	healthy carriage	*Staphylococcus aureus*	ND
2423-1	BY	Placebo	pus	clinical	*Staphylococcus aureus*	ND
2437-3	BN	Placebo	breast milk	healthy carriage	*Staphylococcus aureus*	1
244-1	AJ	Placebo	breast abcess	clinical	*Staphylococcus aureus*	152
2445-5	BL	Placebo	breast milk	healthy carriage	*Staphylococcus kloosii*	NA
2470-9	BK	Azithromycin	breast milk	healthy carriage	*Staphylococcus hominis*	NA
2485-2	CF	Placebo	recto-vaginal swab	healthy carriage	*Staphylococcus arlettae*	NA
2682-4	CG	Azithromycin	recto-vaginal swab	healthy carriage	*Staphylococcus aureus*	ND
2781-3	CA	Placebo	breast milk	healthy carriage	*Staphylococcus aureus*	ND
3005-3	CK	Placebo	recto-vaginal swab	healthy carriage	*Mammaliicoccus fleurettii*	NA
3170-8	CN	Placebo	oropharyngeal swab	healthy carriage	*Staphylococcus aureus*	ND
3176-2	CO	Placebo	oropharyngeal swab	healthy carriage	*Staphylococcus saprophyticus*	NA
3196-8	CS	Placebo	recto-vaginal swab	healthy carriage	*Staphylococcus saprophyticus*	NA
3256-5	CQ	Placebo	oropharyngeal swab	healthy carriage	*Staphylococcus aureus*	152
3285-6	CJ	Placebo	Blood culture	clinical	*Staphylococcus aureus*	ND
3299-5	CR	Azithromycin	oropharyngeal swab	healthy carriage	*Staphylococcus aureus*	15
3387-2	CV	Placebo	recto-vaginal swab	healthy carriage	*Staphylococcus simulans*	NA
349-6	AP	Placebo	Blood culture	clinical	*Staphylococcus aureus*	15
353-9	AL	Placebo	skin swab	clinical	*Staphylococcus aureus*	ND
3532-8	CW	Placebo	recto-vaginal swab	healthy carriage	*Staphylococcus aureus*	8
3549-6	n/a	n/a	n/a	n/a	*Staphylococcus saprophyticus*	NA
3589-6	CY	Placebo	recto-vaginal swab	healthy carriage	*Staphylococcus saprophyticus*	NA
3597-9	CX	Azithromycin	recto-vaginal swab	healthy carriage	*Staphylococcus saprophyticus*	NA
360-4	AM	Placebo	umblical swab	clinical	*Staphylococcus aureus*	672
3622-1	CP	Azithromycin	oropharyngeal swab	healthy carriage	*Staphylococcus aureus*	ND
3634-7	CZ	Azithromycin	recto-vaginal swab	healthy carriage	*Staphylococcus saprophyticus*	NA
3672-5	DA	Placebo	oropharyngeal swab	healthy carriage	*Staphylococcus aureus*	ND
3701-3	CT	Placebo	Blood culture	clinical	*Staphylococcus aureus*	ND
3728-6	DE	Placebo	oropharyngeal swab	healthy carriage	*Staphylococcus aureus*	ND
3748-8	DC	Placebo	breast milk	healthy carriage	*Staphylococcus aureus*	672
3750-2	DC	Placebo	oropharyngeal swab	healthy carriage	*Staphylococcus arlettae*	NA
3751-5	DB	Placebo	breast milk	healthy carriage	*Staphylococcus aureus*	672
3752-8	DA	Placebo	breast milk	healthy carriage	*Staphylococcus aureus*	97
3789-5	n/a	n/a	n/a	n/a	*Staphylococcus aureus*	15
3805-5	n/a	n/a	n/a	n/a	*Staphylococcus aureus*	15
3816-8	DG	Placebo	oropharyngeal swab	healthy carriage	*Staphylococcus aureus*	ND
3881-8	DD	Placebo	breast milk	healthy carriage	*Staphylococcus aureus*	15
3941-7	CD	Placebo	breast milk	healthy carriage	*Staphylococcus aureus*	672
3942-5	CB	Azithromycin	breast milk	healthy carriage	*Staphylococcus saprophyticus*	NA
3954-7	CC	Azithromycin	oropharyngeal swab	healthy carriage	*Staphylococcus aureus*	152
3978-4	DI	Placebo	recto-vaginal swab	healthy carriage	*Staphylococcus aureus*	15
3987-8	DL	Azithromycin	recto-vaginal swab	healthy carriage	*Staphylococcus aureus*	15
4028-9	n/a	n/a	n/a	n/a	*Staphylococcus aureus*	672
4055-8	n/a	n/a	n/a	n/a	*Staphylococcus haemolyticus*	NA
4076-5	DJ	Azithromycin	breast milk	healthy carriage	*Staphylococcus aureus*	5
4087-3	DC	Placebo	breast milk	healthy carriage	*Staphylococcus aureus*	672
4129-1	DO	Placebo	recto-vaginal swab	healthy carriage	*Staphylococcus aureus*	15
4179-3	DM	Placebo	breast milk	healthy carriage	*Staphylococcus aureus*	6
424-3	AN	Placebo	eye swab	clinical	*Staphylococcus aureus*	15
4325-7	DQ	Placebo	breast milk	healthy carriage	*Staphylococcus aureus*	672
4335-8	DR	Azithromycin	oropharyngeal swab	healthy carriage	*Staphylococcus aureus*	30
4341-3	DH	Azithromycin	breast milk	healthy carriage	*Staphylococcus aureus*	669
4345-1	n/a	n/a	n/a	n/a	*Staphylococcus aureus*	2498
4362-7	DS	Azithromycin	oropharyngeal swab	healthy carriage	*Staphylococcus aureus*	15
4454-3	DT	Placebo	oropharyngeal swab	healthy carriage	*Staphylococcus aureus*	ND
456-4	CL	Placebo	Blood culture	clinical	*Staphylococcus aureus*	30
4560-9	DP	Placebo	breast milk	healthy carriage	*Staphylococcus aureus*	15
4679-7	DU	Azithromycin	oropharyngeal swab	healthy carriage	*Staphylococcus aureus*	152
473-4	AC	Placebo	breast abcess	clinical	*Staphylococcus aureus*	852
481-4	AO	Placebo	Blood culture	clinical	*Staphylococcus aureus*	852
482-3	AB	Azithromycin	eye swab	clinical	*Staphylococcus aureus*	5
51-5	AA	Placebo	skin swab	clinical	*Staphylococcus aureus*	15
520-9	AB	Azithromycin	ear swab	clinical	*Staphylococcus aureus*	5
5521-6	DV	Azithromycin	breast abcess	clinical	*Staphylococcus aureus*	152
57-7	AE	Placebo	eye swab	clinical	*Staphylococcus aureus*	15
617-9	AR	Placebo	skin swab	clinical	*Staphylococcus aureus*	152
648-3	AY	Azithromycin	umblical swab	clinical	*Staphylococcus aureus*	ND
671-2	AQ	Placebo	skin swab	clinical	*Staphylococcus aureus*	15
693-4	AS	Placebo	skin swab	clinical	*Staphylococcus aureus*	152
713-8	AT	Placebo	umblical swab	clinical	*Staphylococcus aureus*	5
738-9	AV	Azithromycin	Blood culture	clinical	*Staphylococcus aureus*	8
755-7	AW	Placebo	skin swab	clinical	*Staphylococcus aureus*	5
774-1	AU	Placebo	Blood culture	clinical	*Staphylococcus aureus*	672
826-1	AX	Placebo	umblical swab	clinical	*Staphylococcus aureus*	ND
893-8	AZ	Placebo	skin swab	clinical	*Staphylococcus aureus*	15
918-5	BA	Azithromycin	oropharyngeal swab	healthy carriage	*Staphylococcus aureus*	ND
98-5	AG	Azithromycin	umblical swab	clinical	*Staphylococcus aureus*	ND

The patient column represents random 2-letter identification codes, not patient initials, n/a - not available
