# Supplementary material for: Genomic approach to determine sources of neonatal Staphylococcus aureus infection from carriage in the Gambia
Source: BMC Infect Dis. 2024 Sep 9;24:941. doi: 10.1186/s12879-024-09837-5 (PMC11384681; doi:10.1186/s12879-024-09837-5)
Supplement: Supplementary file 5 — Supplementary Material 5 [file 12879_2024_9837_MOESM5_ESM.pdf]

Suppl. Table 2. Distribution of *S. aureus* Sequence Types by Sample Type and Source

		Sequence Types													
**Subject**	**Sample types**	**ST1**	**ST5**	**ST6**	**ST8**	**ST15**	**ST30**	**ST97**	**ST121**	**ST152**	**ST669**	**ST672**	**ST852**	**ST2498**	**ND**
**Mother**	Oropharyngeal swab	1	1	0	0	4	1	1	0	3	0	1	0	0	10
	Breast milk	3	2	1	0	3	0	1	0	0	1	5	1	0	4
	Recto-vaginal swab	1	1	0	1	6	0	0	1	0	0	1	0	0	2
**Baby**	Oropharyngeal swab*	0	0	0	0	19	0	0	0	0	0	5	8	0	11
	Eye swab	0	3	0	0	2	0	0	0	0	0	0	0	0	0
	Umbilical swab	0	1	0	0	1	0	0	0	0	0	1	0	0	3
	Blood culture	0	0	0	1	2	1	0	0	0	0	1	1	0	2
	Pus	0	0	0	0	0	0	0	0	0	0	0	0	0	1
	Abcess	0	0	0	0	0	0	0	0	2	0	0	1	0	0
	Skin swab	0	1	0	0	3	0	0	0	2	0	0	0	0	1
	Ear swab	0	1	0	0	0	0	0	0	0	0	0	0	0	0

*up to 8 suspected colonies of *S. aureus* were screened from the same sample

ND - Not Determined (sequence type could not be determined due to missing allele number(s))
