# Supplementary material for: Genomic approach to determine sources of neonatal Staphylococcus aureus infection from carriage in the Gambia
Source: BMC Infect Dis. 2024 Sep 9;24:941. doi: 10.1186/s12879-024-09837-5 (PMC11384681; doi:10.1186/s12879-024-09837-5)
Supplement: Supplementary file 6 — Supplementary Material 6 [file 12879_2024_9837_MOESM6_ESM.pdf]

Supplemental Information

Suppl. Table 1. List of staphyloccoci species among study participants.

Suppl. Table 2. Distribution of *S. aureus* Sequence Types by sample type and source

Supplemental Figure 1. Selection process for clinical and carriage samples from the PregnAnZI-2 trial. A total of 172 *S. aureus*-positive samples were randomly selected. Colored boxes indicate when isolates were obtained from oropharyngeal swabs collected from the same newborns from which other anatomical sites were sampled.

Supplemental Figure 2. Maximum likelihood phylogenetic tree showing the evolutionary relationship between Gambian ST15 and a global collection of 1157 public ST15s.

Supplemental Figure 3. Dot plots showing the association between *S. aureus* STs and the different sample types. The size of the dots represents the number of samples that corresponds to each ST identified.
